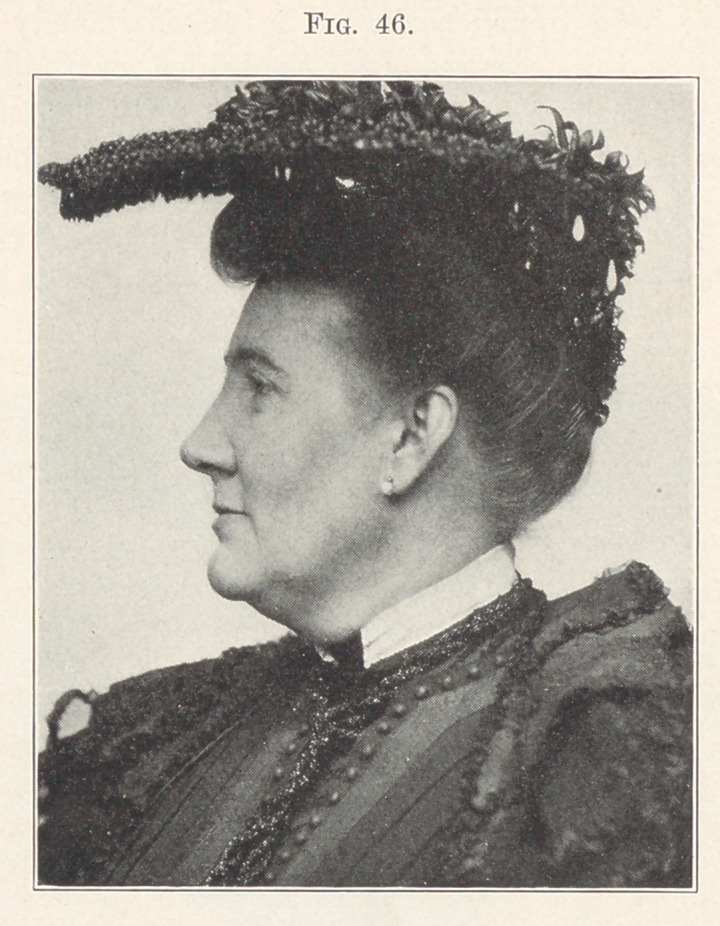# Some Basic Principles in Orthodontia

**Published:** 1903-10

**Authors:** Edward H. Angle

**Affiliations:** St. Louis, Mo.


### International Dental Journal.

VOL. XXIV. OCTOBER, 1903. NO. 10.

### Original Communications.[1]

### SOME BASIC PRINCIPLES IN ORTHODONTIA.[2]

BY EDWARD H. ANGLE, M.D., D.D.S., ST. LOUIS, MO.

MR. PRESIDENT AND MEMBERS OF THE NEW YORK INSTITUTE OF STOMATOLOGY,—It is with much pleasure that I meet with so prominent an organization to-night for the purpose of considering some phases of that important and fascinating branch of science known as orthodontia, a science pregnant with greatness, and yet so feebly taught in our colleges and so little appreciated and imperfectly practised by our profession that it still lingers on the threshold of its limitless possibilities. Indeed, it is doubtful whether its general practice up to date would not show a balance on the side of harm rather than of benefit. Yet the opportunities it affords for physically benefiting humanity are so great that all intrusted with the care of the teeth may well devote much time to its consideration. And let us not pass lightly over this phrase "physically benefiting humanity." To detect the marring effect

[1] The editor and publishers are not responsible for the views of authors of papers published in this department, nor for any claim to novelty, or otherwise, that may be made by them. No papers will be received for this department that have appeared in any other journal published in the country.

[2] Address given before The New York Institute of Stomatology, October 7, 1902.

of maloccluded teeth and cramped and diminished arches on the facial lines, often even to the extent of repulsiveness, is not difficult; but who can estimate the effect on the health and usefulness of the individual, on the breathing apparatus, on the function of mastication, and on the voice in speech and song? And these deformities are not limited merely to an occasional unfortunate, but are widely distributed and vast in numbers. Yet they all have beginnings and are all progressive. Intelligent treatment at the proper time would correct abnormal tendencies, and nature would complete the development of the dental apparatus in harmony with the requirements of utility and beauty. Truly this great subject demands our most thoughtful attention.

I shall try this evening to make clear some principles which seem to me basic, and on the intelligent comprehension and application of which depend the possibilities of successful achievement.

First, I shall hope to demonstrate to you that we must consider the dental apparatus as a whole *in each case,* together with the throat and nose and facial lines, instead of limiting our attention to local symptoms in the form of one or more crooked teeth, as has so long been the practice.

Secondly, I shall try to impress you from the orthodontist's stand-point with the value of each individual tooth and with the absolute necessity of preserving the full complement of teeth, or its equivalent, in every case. I shall try to bring conclusive evidence that the sacrifice of teeth for either the intended prevention or correction of malocclusion is not only wrong practice and fallacious teaching, but most baneful in its results. I shall further try to show that the full complement of teeth is necessary to establish the most pleasing harmony of the facial lines.

Thirdly, I shall try to prove to you that the first molars are the most important of the teeth, and that they are the first to be considered, from the orthodontist's stand-point, in both diagnosis and treatment; that we must first look to their correct adjustment instead of beginning with the incisors and ignoring the positions of the molars, or attempting to correct them last.

Fourthly, it is positively essential that each arch and the teeth of each arch shall receive at least equal care in their adjustment, the preference, if any, being given to the lower.

And lastly, I shall try to show you that fully ninety per cent.

of the regulating appliances represented in our literature are constructed and operated upon incorrect principles.

I shall not have time to touch upon the etiology of malocclusion, but I feel that I should not miss this opportunity to say that I believe it is as ignorant as it is cruel to brand as degenerates those suffering from malocclusion of the teeth.

I hope I shall this evening awaken much interest, and if we do not agree on some points it will not be the first time that men have differed on the subject of orthodontia, yet I hope and believe that our differences will be honest.

The picture now upon the screen (Fig. 1) represents a type of malocclusion almost as familiar to you all as the signs on your

Fig. 1.

office doors. Again and again have you seen such a case in some of its variations. It amounts to nearly seven hundred in a thousand cases of malocclusion. It has become so common to you that few of you realize how seriously the person afflicted therewith is handicapped. The function of mastication is seriously impaired, and what would the eloquence of a Demosthenes avail a person with such teeth, for he could not enunciate one word in ten correctly; or what would an endowment of vocal gifts equal to those of Patti profit a person with vault and arch so abnormally constricted as to render the highest cultivation of the vocal chords under the best teachers of little avail? And as to its effect on the beauty of the face, no matter how beautiful the eyes, faultless the nose, lovely the skin and complexion, or otherwise perfect harmony of proportion of the features, all is counterbalanced by this pronounced blemish. How mightily would your opinions change as to the wonderful beauty of the Venus de Milo if she should smile and reveal such shocking dental chaos. Beautiful eyes, excellent proportions of the features, even a pleasant smile,—all together cannot offset this unfortunate blemish, as is proved by the next picture (Fig. 2), but, on the contrary, I sometimes think intensifies it.

Fig. 2.

Who can estimate the effect such a blemish plays in the mental development of children so affected, especially if they have reflective, sensitive natures, as is frequently the case? Now, there was a time in the early developmental stages of this case when the irregularities were but slight and could have been easily corrected, but the parents of the child were told, as dentists have long been in the habit of telling parents, that the patient was too young for treatment, and so it progressed as countless thousands all over this city—all over this broad land—do progress into similar deformities, under the very eyes and with the advice of even the best dentists.

Let us note the progress of this type of malocclusion, tracing its development through several cases at different ages to complications equal to those just described.

But before doing so let me freshen your memories and fix in your minds, as clearly as I am able, the picture of normal occlusion which is the very basis of all our efforts in orthodontia, as well as in all dentistry, and yet I am sorry to say that only a very small percentage of dentists (probably less than three per cent.) know the correct occlusion of the teeth individually or collectively. (If you doubt this, quiz a few of your dental friends on the subject.)

You will remember that the case just shown belongs to Class I. The first molars were locked in their normal mesio-distal relations at eruption, and the malocclusion limited to the teeth anterior to the first molars—principally to the incisors. Let us note the marvellous contrast in the next picture (Fig. 3), which typifies normal

Fig. 3.

occlusion and the normal relations of the occlusal planes; and we know that it means more than this, for it also means normal tongue space, normal respiration, normal balance of lip pressure and function, for without all these we could not have such ideal occlusion. This picture is one that I wish you to carry with you and compare with each and every other picture that you will see upon this screen to-night, for it represents the normal, and without a clear conception of the normal we cannot possibly have a true conception of the abnormal in occlusion. We could study and discuss this marvellous picture for a full hour, for it is very rich in graceful, artistic curves, forms, proportions, contrasts, and effects, as well as wonderful in mechanics and correlation of forces.

This normal occlusion is maintained only through the normal relations of the inclined planes of the cusps, assisted by the normal influence of the muscles exercised externally and internally upon the crowns of the teeth.

There are two points of great importance in the occlusion that I also wish you to remember. First, the normal relations of the first molars, and second, that of the cuspids. The first determines the mesio-distal relations of both lateral halves of the arches; the second, the width of the arches. If the first molars lock normally, as you see in this picture, the mesio-buccal cusp of the upper will occlude in the buccal groove between the mesio- and disto-buccal cusps of the lower. And if the first molars are so locked in their eruption it will make *possible* the normal eruption of *all the teeth* both anterior and posterior to them, as has resulted and is here shown in this beautiful picture. But if the first molars lock mesially to normal or distally to normal in their eruption, it will *necessitate* the eruption into positions of malocclusion of all the remaining teeth both anterior and posterior to them, and according as these molars erupt and lock in mesial or distal relations, in one of the lateral halves of the arches, or both, will be determined certain classes of malocclusion which will be considered later.

Now, if the locking of these molars plays so important a part in the eruption and positions of the remaining teeth, can you not see how important it is that they be preserved and early attention given to their eruption and relations? Hence the time for beginning treatment of malocclusion is no longer mythical, but as fixed and well-defined as the first molars themselves.

It must be borne in mind, however, that even with the normal locking of the first molars and normal mesio-distal relations of the jaws and arches, the normal locking of all the other teeth is by no means assured, and malocclusion may involve any or all of the teeth anterior to them, but usually is chiefly confined to the narrowing of the arches in the region of the cuspids, with bunching of the incisors, similar to the case first illustrated, and, as we have said, by far the largest number of cases of malocclusion belong to this class, and it is to this great class we will first direct our attention.

Before leaving this picture let me try to impress you with the importance and wonderful relations of the occlusal planes,—how that we must gain their normal relations if we would hope to be successful in maintaining them in the positions in which we wish them to remain after correction. And what a waste of time to consider one arch without the other, or to attempt to ignore the importance that each tooth bears to all other teeth in both arches. Or, in other words, this picture of normal occlusion and all that it means must actuate and direct all of our efforts from the beginning of treatment to the termination of retention.

Let me also try to impress you, before leaving this picture, that nature could never unaided have effected such perfect occlusion had there not been also normal lip pressure and normal respiration. So there are two forces acting upon the teeth, either one of which might disturb the balance of harmony in the development of the jaws and eruption of the teeth, and to work in ignorance of these forces and to direct our efforts to the symptoms only, manifested in so-called "crooked teeth," is a plan that intelligent dentists ought henceforth to abandon.

I shall not have time to consider in this lecture as fully and as thoroughly as I should like the relations that occlusion bears to the contour of the face. I hope to discuss this phase of the subject to-morrow at the meeting of the American Society of Orthodontists in Philadelphia, a full report of which will appear in the *Items of Interest.* Let me here say, however, that as we know that all of the teeth are essential to the best occlusion, equally as important is it to the most pleasing contour of the face.

The picture now upon the screen (Fig. 4) shows a face of most beautiful proportions. There can be no doubt that all of the teeth are present and in perfect occlusion. Such beautiful proportions could not exist with anything short of the full complement of teeth which were erupted and locked normally.

The next picture (Fig. 5) represents the case of a healthy child aged eight years. The first molars have locked normally. The permanent central and lateral incisors are erupting normally—plenty of room for all; but the next picture (Fig. 6) will show a far more common condition. Although the molars have locked normally, insuring the normal mesio-distal relations of the two arches, there is a lack of space for the eruption of the centrals and laterals. You will note that the lower incisors erupt first. The arc which they are forming is the mould over which the arch for the erupting upper teeth will be formed. Please bear this in mind: unless the lower incisors take their correct positions the upper ones cannot possibly take their correct positions. How important, then, that the lower incisors be mechanically assisted, if necessary, to gain and maintain their correct positions.

FIG. 4.

FIG. 5.

The first picture you saw upon the screen to-night had a beginning probably very nearly like the case here represented.

Fig. 7 represents a similar case in which I corrected the positions of the lower incisors early, establishing harmony in the relations of the two arches and thus removing all obstructions for the remaining teeth that were to erupt.

Fig. 6.

Fig. 7.

Fig. 8 represents another case in which the eruption of the incisors had been allowed to progress still farther. The lower incisors are greatly bunched, one of them being deflected entirely lingual to the normal line of occlusion, as you will see in the next picture (Fig. 9).

There could be no better illustration of how the upper teeth are forced by reason of lip pressure into abnormal positions in being moulded over the abnormally small arc of the lower. All of this is simply the result of mechanics,—a slight deflection at first from the normal position, which made possible and easy the continuation of this deflection of the erupting teeth from their natural positions. Had the lower incisors been early corrected, the results here

Fig. 8.

shown could not have taken place, and yet it is common to have such a condition explained by the very wiseacres as being the result

Fig. 9.

of heredity, the child inheriting the large teeth of one parent and the small jaws of the other. It is time that such nonsense passed out of the minds of intelligent dentists. Nature does not make such mistakes in so important a matter as the dental apparatus.

Fig. 10.

It is time also for intelligent dentists to know that extraction in this case, and in all such cases, to gain room for the crowded teeth is an evidence of ignorance and is as pernicious as it is unneces-

Fig. 11.

sary. There is an abundance of room for the teeth in these arches, as in all cases of this kind, as is well shown in the next picture (Fig. 10), where all the teeth have been moved into the line of occlusion, and when the two models are placed together, as shown in Fig. 11, you will see that there is harmony in the sizes of the arches and in the occlusal planes, each one giving support to all the others. These occlusal planes will come more and more into

FIG. 12.

harmony as the teeth settle into their places, and I want to show you that the lips have not been crowded out to the size of a Hottentot's, either, but only to the extent that nature intended they should be to give the best and most pleasing contour to the face, as you will see by the next picture (Fig 12), and I would ask you if this boy looks like a degenerate.

Let us take another case, Fig. 13, the same in principle as all those we have seen, only it has been neglected a little longer and malocclusion has developed a little farther. We know that it belongs to the same great class of cases, for we see that the molars are normally locked, and we know the mesio-distal relations of the arches must be normal, the same as all those cases we have thus far considered. Had this boy received treatment early in the eruption of his incisors, this pronounced deformity might easily have been prevented, but his parents were repeatedly told by dentists that he was too young; that he should wait until all his teeth were in place before having anything done, and that probably nature would correct their positions. You see the result. Other dentists wanted to extract, and that is just what I would have thought necessary a few years ago, but now I know it would have been a most inexcusable blunder, and I see or know of many such blunders being daily performed. What is clearly indicated is to correct the occlu-

Fig. 13.

*sion—to place each occlusal plane in its harmonious relations with its opposing occlusal planes,* just as nature intended it to be.

The next picture (Fig. 14) shows where this has been accomplished, and you see there is an abundance of room in the alveolus for all of the teeth. Of course, this was not true immediately after the movement of the crowns of the teeth into their correct positions, for the alveolus had been arrested in its development. It had only been developed, as it must always be, in accordance with the positions in which the teeth arranged themselves, and as soon as they were placed in their correct positions nature was stimulated to complete the development of the alveolus, and you will note how pronounced this was in the two years that intervened between the time in which they were moved into their correct positions and the time when this model was made.

Let us examine another case, Fig. 15, belonging to this same

FIG. 14.

FIG. 15.

great class, where malocclusion has been permitted to progress unchecked still farther. Surely here, you will say, the jaws are too small for the teeth, and extraction must be resorted to. But it was only necessary to enlarge each dental arch sufficiently to move the crown of each tooth into its correct position in the line of occlusion and retain it there until nature could complete the development of the alveolus, as shown in the next picture (Fig. 16). There

Fig. 16.

was then harmony in the sizes of the arches and each gave support to the other through the correct relations of the occlusal planes, and the excellent results in the facial lines are shown by the next picture (Fig. 17).

The discovery of the fact that nature will build in a sufficient amount of alveolar tissue to meet the requirements of the teeth in their new positions, and restore the lack of contour to the face, is of inestimable value to the science of orthodontia, yet this fact would never have been known without accurately made models from plaster impressions, from which we can easily determine the extent of the growth of bone; and I wish to here make protest against the slovenly, inaccurate, unscientific models that are usually shown as evidence in such cases. You know that it is impossible for any one to take an accurate impression of the teeth and alveolus with any of the wax or plastic materials, and models made from such impressions ought no longer to be accepted as evidence, for at best

FIG. 17.

they serve only as a base of conjecture. Models should be made with the same degree of skill that would be exercised in making the finest fillings, bridges, or dentures. When placed in articulation we should be able to examine them from every point, that we may study carefully the relation of each occlusal plane, the position and direction of each root, as shown by the next picture (Fig. 18). Such models are of lasting scientific value, and a collection of them forms the most valuable library of orthodontic literature.

I should like to show you many more models belonging to this

Fig. 18.

great class, and the accurate measurements from which have been determined how much the very apices of the roots have been shifted by

Fig. 19.

nature as a result of establishing normal occlusion of their crowns by means of the regulating appliances, but I must hasten to another great class, well defined and unmistakable if you have but learned to diagnose it properly, beginning the diagnosis with the first molars instead of looking at the incisors alone, for I must repeat that in this class the positions of the incisors are but the symptoms, the relations of the molars the cause (not the primary cause).

The two models now shown upon the screen (Fig. 19) illustrate well the main principles of all cases you will ever find that belong to this great class, which I have named Class II. They represent the two divisions of this class. They are alike in the main, for the lower first molars in both cases erupted and locked in distal occlusion on both sides,—that is, one cusp distal to normal,—which necessitated all of the teeth anterior and posterior erupting and locking in distal or abnormal relations. The remarkable difference which the incisors in the two divisions present is due to the difference in the lip functions. The case in which the incisors protrude results from the lack of lip function, the patient being a mouth-breather and the upper lip being elevated and exercising little or no pressure on the labial surfaces of the incisors, allowing them to move outward. This outward movement has been intensified by the lower lip being constantly forced behind them in the effort to close the mouth. In the other model, the bunched and flattened positions of the incisors are due to the influence which the lip has exerted upon them, for such patients are normal breathers, keep the mouth closed the requisite amount of time, and the normal, well-developed lip exercises a strong pressure upon them, thus bunching them as they are forced back to meet the lower incisors.

The two pictures now upon the screen (Fig. 20) show the remarkable contrast in the faces of the patients whose malocclusion was represented by the models you have just seen, and you will please note how different the development of the nose and lip of the normal breather is from that of the buccal or mouth-breather, and also how the facial lines of both are thrown out of balance by the recession of the mandible, due to the teeth being in distal occlusion. Had the first molars been watched during their eruption and locking, and forced by proper mechanical means to assume normal relations, and there retained with suitable devices, a very different result from that which you see here must have followed, especially if the throat and nose of the buccal breather had also been looked after and successfully treated.

In the treatment of these cases I believe I can again prove to you that my theory is correct, that extraction is wrong, that the full complement of teeth is necessary to the best results, and that each tooth shall be made to assume its correct relation with its fellows. In other words, if the molars and premolars of the upper

Fig. 20.

dental arch be moved distally one-half the width of a cusp of a molar or premolar, and the molars and premolars of the lower arch be tipped forward in their alveoli to the same extent, or one-half the width of a cusp of a molar or premolar, there will then be normal mesio-distal relations of these teeth, and if the arches in the region of the incisors be put in true at the same time, there will be harmony in their relations and the best effect will have been produced upon the facial lines. In other words, we will have established normal occlusion with *all* its possible benefits.

This plan of treatment I have been practising now but three years, and so pleased am I with it in the large number of cases that I have so treated that I no longer practise or believe in the plans that I formerly advocated, or that of gaining harmony in the sizes of the arches by the sacrifice of the two first premolars in the upper arch and retracting the cuspids and incisors to close the spaces, or by the plan known as "jumping the bite," first advocated by my friend, Dr. Kingsley, consisting of first placing the teeth of each arch in correct alignment and then compelling closure of the mandible forward the width of one premolar tooth on each side, so that all of the teeth were in normal occlusion. That both of these plans may and have been more or less successfully followed there can be no doubt, but I believe them to be far more tedious, more difficult of accomplishment, and more uncertain as to satisfactory results than the plan I now follow.

I regret that I have not time to show you a large number of cases belonging to the two divisions of this great class, embracing a wide range as to age and stages of development, but must content myself with only a few cases typical of each.

The next picture (Fig. 21) will show you the models of a very well defined case belonging to the second division of this class. The

Fig. 21.

patient was a young man twenty-two years old, of massive frame, large jaws, and large teeth firmly set in their alveoli. He was a normal breather. As you will observe, there was complete distal occlusion of the teeth in both lateral halves of the arches, with bunched and retruding upper incisors and a very weak expression in the lower part of the face, due to this condition.

Now, as to treatment. The upper molars and premolars were moved distally and the lowers mesially until they were in normal occlusion, as shown in the next picture (Fig. 22). You will see that each occlusal plane is in normal relation with its opposing occlusal plane, thus locking and assisting in its retention, and I assure you that the facial lines were as greatly improved as was the occlusion.

And how was this accomplished? you will naturally ask, for you must justly reason that to move all of the teeth in both arches, as has been done, certainly would require a considerable degree of force, and that it should be directed in the right direction.

So far I have said nothing about regulating appliances, for I have wished to impress you with principles more important. I believe regulating appliances have heretofore been made far too prominent in such discussions—have been put far in advance of a comprehension of the principles which should go first and actuate their intelligent construction and operation. They have been exploited until their name is legion. They represent about all that there is in mechanics. They may justly be said to typify ingenuity,

Fig. 22.

as well as complexity, crudity, and absurdity. Had their designers and makers understood the importance of occlusion I believe there never would have been the hundredth part of the number that now burdens our literature to confuse the student and torture humanity.

I formerly advocated a few combinations of appliances which I have now largely abandoned. I believed the headgear and chin retractor were valuable. The latter is now entirely obsolete in my practice and the former but rarely used, and the same might be said of the traction screw and rotating levers. The jack-screw has been and doubtless will long continue to be the one form of regulating appliance most used by dentists, for it seems almost impossible to get dentists to study occlusion, its bearing upon and importance to orthodontia, but they can and do reason only from the basis of the mere symptoms, or "crooked teeth," as they call them, and they naturally reason that a jack-screw placed against a tooth that seems to be "straight" and made to operate at its other end against one that is "crooked," to push it into a better position, is the one thing needful, but I believe the jack-screw to be one of the poorest of regulating appliances, and I say this notwithstanding that I am the inventor of what I believe to be the most simple and efficient one yet brought out, and one that has more base imitations than any other of my inventions. But I now think the principle is wrong with the jack-screw, as it is with all those forms of appliances that are made to act locally, so to speak, or upon only the teeth that seem "crooked," instead of one that shall be operative from the basis of occlusion, having the control of one or of all of the teeth of not only one but of both arches, if need be. I cannot bring out the point too forcibly that it should be our mission to improve the dental apparatus as a whole through occlusion, for in this way only can our efforts be fruitful of the best results in not only bettering the principal function of the teeth,—mastication,—but their appearance, as well as giving greater freedom to the movements of the tongue, and also making possible the modification of the vault of the arch towards the more normal growth and development of the nasal tract, and last, but of great importance, a better contour of the face with more pleasing lines of facial expression.

I am now accomplishing fully ninety-eight per cent. of the tooth movements in my practice with but a single appliance, and performing them far easier and more quickly than I ever did with all the various combinations I have ever advocated in the past, which at most were very few, for it has ever been my aim to simplify both the diagnosis and treatment of cases in my practice, and all of the cases you will see on this screen to-night have been treated with but one appliance,—namely, the expansion arch,—and although I believe that I have added some valuable improvements to it, yet it was known and used before this republic was. It was first used by that greatest of the early dentists, the Frenchman Fauchard.

The next picture (Fig. 23) shows it as I now use it. In temper it contains much spring, sufficient to speedily widen the dental arch, if need be, and having self-locking nuts to properly adjust it to the demands of expansion. It is round instead of being half-round or flat, as used by the older writers, which better prevents the accumulation of food, as well as making it more compact and less conspicuous. My latest improvement to it is a delicate rib on the periphery of the unthreaded portion. This is to be notched at desired points to prevent the slipping of wire ligatures, this form of ligature being not only a very valuable addition to orthodontia, but making this wonderful appliance vastly more efficient. I have called it wonderful, and truly it is, and he who intelligently experiments with it will grow daily more and more impressed with its great possibilities in correcting malocclusion. In my opinion there

FIG. 23.

is no tooth movement, be it simple or complicated, that cannot be performed more quickly and easily with this than with any other device, and I have arrived at this conclusion not hastily, but gradually, and one by one have abandoned nearly all of the other once favored appliances.

With it we not only have complete control of the direction of the teeth we wish to move, but all others that we wish to prevent moving or to enlist as anchorage either in the form of simple, stationary, or reciprocal anchorage, etc., and you all know how important a part anchorage plays in tooth movement.

Let us study for a moment the use of the arch in a severe test in complicated tooth movements where all of the teeth are to be moved, as shown in the next picture (Fig. 24), for the dental arch must be widened, the laterals and centrals moved forward and rotated. All was accomplished at the same time. You will please leave out of consideration the reinforcement spring shown in the vault of the arch, for this is only an auxiliary and may or may not

Fig. 24.

be used, but is only rarely necessary, and you will see what perfect control is gained over each tooth, and how the force from the tightening of the unflinching ligatures, from the spring of the arch, and from the nuts in front of the tubes on the anchor teeth is distributed and reciprocated. I wish we might spend much more time on this picture, but I have carefully described it in other writings,[1] and must hasten to other pictures, only stopping here to say that in all cases belonging to the first class, if we have used this appliance intelligently and have adjusted each tooth in each arch, the arches and the occlusal planes will then be in harmony, and if the teeth are in distal occlusion, as in the case considered but a few moments ago, the lower teeth may be easily shifted mesially and the upper teeth distally into harmony of occlusion, it only being necessary to use two of the expansion arches, and reciprocating the force from one to the other, as shown in the next picture (Fig. 25), the force being derived from one or more delicate rubber ligatures made to engage the distal ends of the tubes of the bands on the anchor teeth of the lower arch, and sheath-hooks which have been attached at desired points to the upper expansion arch. By studying this picture carefully you will see that force is exerted in the exact direc-

[1] Treatment of Malocclusion of the Teeth and Fractures of the Maxillæ, sixth edition.

tion it is needed, and at the same time most inconspicuously and with very little inconvenience to the patient.

Fig. 25.

When you consider what a large percentage of cases there are belonging to this class and how easily and successfully they may be treated by this method, you must be impressed with the fact that a most valuable step forward has been gained in the science of orthodontia. Now, I know that when anything new and valuable is brought out in dentistry there is usually that familiar type of individual who will rise up and say, "Why, I have been using that for twenty-five years," but to my mind this savors of "degeneracy." The fact is, to the best of my knowledge and belief we are indebted to Dr. H. A. Baker,[1] of Boston, for this idea, he having used it in the retraction of the protruding incisors of his son a number of years ago, and it was from him I received the idea. I have hence called it the "Baker anchorage," and it has almost revolutionized my daily practice. In its use, however, I would add this important improvement,—that the force be directed upon the molars first, instead of on the incisors, their positions being, as I believe, merely the result of the malpositions of the molars, and we should unravel the complexities of these cases by beginning right, that is, with the molars, following with the premolars, and lastly adjusting the incisors. And using it as here shown, the

[1] Since giving the above address I have learned that Dr. Calvin S. Case, of Chicago, also employed this form of anchorage, probably at about the same time as Dr. Baker. It is reported in the Transactions of the Columbian Dental Congress.

force is directly received upon the first molars, pushing the uppers distally and pulling the lowers mesially. Of course, all the lower teeth, as here shown, will be carried forward, and all the force required in their movement will be pitted against the upper first molars. As these move distally (the nuts being occasionally tightened), more or less space will be noted between them and the second premolars, and after the molars have been carried well back into correct positions the anchor bands should be removed and similar, smaller bands (X bands) placed upon the second premolars and the expansion arch again applied. Wire ligatures are also made to engage both first and second premolars on each side, and force from the rubber ligatures again exerted. After the premolars are well back into position the nuts in front of the tubes on the anchor teeth are loosened, or removed entirely, allowing the force of the rubber ligatures to be received upon the incisors through the centre of the arch. In this way the incisors, if they be prominent, are soon retracted.

It is needless to say that care, judgment, and skill are necessary to successfully—and, I may add, almost painlessly—operate this device, and the progress made in these cases should be easy, speedy, and continuous, but with the careless and unskilful much trouble and annoyance is but natural.

Of course, it is of the utmost importance that the teeth shall be mechanically retained in their new positions. The real retaining devices are the inclined occlusal planes, but these must be assisted for a time by a mechanical device, or of course the teeth that have been moved will speedily revert to their original positions, and the next picture (Fig. 26) will show you a simple device for holding the teeth that have been moved mesially in the lower arch and those that have been moved distally in the upper arch in normal relations. I have been using this with much success for a long time. At first I used a spur cemented into a tooth,[1] but later attached the spurs to accurately fitted clamp-bands, the spur being made to close in front of a metal plane attached to a band upon an opposing tooth, as you now see them. They may be used either upon molars or premolars. The bands must be accurately fitted and carefully cemented, and the plane and spur correctly placed. If this

---

[1] Regulation of Teeth and Treatment of Fractures of the Maxillæ, fourth edition.

be properly done they will last as long as desired. I have had them remain in position two years without loosening, but unless they

Fig. 26.

are properly adjusted they will give trouble, the one usually giving way being the spurred band.

Fig. 27.

Fig. 28.

One word of caution, for this picture is misleading. It represents the spur of considerable length. It should be short, only sufficient in length to pass beyond the end of the metal plane when the mouth is closed. The edges of the bands should also be close to the occlusal margins of the teeth, and the plane of metal close to this edge of the band. This is important, for it makes possible a very short spur, and consequently far less strain upon the band than when a long lever-like spur is used.

The other devices shown in the cut are for the retention of incisors and cuspids, and are so well known that I will not take up your time here with an explanation of them.

The next picture (Fig. 27) will show another case belonging to the same class we were last discussing, only this patient is much younger, and you can easily imagine how much more quickly and easily the teeth were adjusted to the positions shown in the study model, Fig. 28. The retaining device, as just described, is also shown here.

Fig. 29 shows a most pronounced case of this type, and you will note how greatly the bite is shortened in connection with the

FIG. 29.

bilateral distal occlusion. How unfortunate it is that the adjustment of the first molars could not have been accomplished early, to prevent this condition. No wonder the face both in front and in the profile, shown in Fig. 30, shows such a shortening and inharmony of contour, nor that it is so vastly improved after treatment, as shown in Fig. 31. The occlusion after treatment is shown in the study model, Fig. 32. I ask you what would have been the effect on the facial lines if extraction had been resorted to in this case?

Cases belonging to the subdivision of this division of this class are of course numerous. Fig. 33 shows one that is typical, or uni-

Fig. 30.

Fig. 31.

FIG. 32.

FIG. 33.

FIG. 34.

laterally distal, and Fig. 34 represents the case after the shifting distally of the left molars, premolars, and cuspid of the upper arch, as well as the correction of the positions of the incisors, and the shifting mesially of the left molars, premolars, and cuspid of the lower arch was accomplished. You will notice how the length of overbite of the incisors has also been improved, as it must be in all such cases if this plan of treatment be followed.

The next picture (Fig. 35) shows a most pronounced case belonging to the first division of this class, or the buccal-breathing and protruding-incisor type, and notwithstanding that there is such great prominence of the incisors, yet the molars and premolars occupy the same positions as in all this great class, so there would be no more excuse for extracting two premolars from the upper arch in this case than there would be in the ordinary case of far less protrusion.

The next picture (Fig. 36) shows a study model of the occlusal surfaces of the upper teeth, and you will see by the spaces posterior to the cuspids how much the molars and premolars have been carried distally, and the next study model, Fig. 37, shows the corrected occlusion, although the teeth with their retainers have not yet settled into position sufficiently for the making of final, perfect models of the case.

I could show you a large number more, but they would all resemble this so closely in form and in detail of treatment that it would be but mere repetition. I will only add that in all these cases where I have sacrificed premolars I believe I have blundered, but this was before we had the Baker anchorage. Now there seems to be no longer excuse for such extraction.

Fig. 38 illustrates a case typifying a subdivision of Division 2, Class II., or unilateral distal occlusion. These subdivisions are perhaps more numerous than the parent type, and their treatment in so far as the malocclusion exists is identical with that described for cases bilaterally distal, the Baker anchorage being used only on the maloccluded side.

Fig. 39 represents the case after having been so treated and harmony of the occlusal planes established.

There is not time this evening to fully consider cases belonging to the third great class of malocclusion. I will show but one,—that of a comparatively young person,—in Fig. 40.

I believe that if we begin treatment early, retain all of the

FIG. 35.

FIG. 36.

FIG. 37.

FIG. 38.

FIG. 39.

FIG. 40.

teeth, shift the first molars into normal occlusion and lock them there, we will have accomplished the best results possible. Fig. 41

Fig. 41.

shows this case only three weeks after treatment was commenced. I assure you that the rapidity with which the teeth moved in this case was as great a surprise to me as to the patient. The teeth were retained for about six months, when they had settled into very ideal relations.

The plan of treatment was the same as that described for cases belonging to Class II., only, of course, the direction of force was reversed, as shown in Fig. 42.

Fig. 42.

Cases belonging to the subdivision of this class are so rare it is not necessary here to discuss them.

So far I have tried to impress you with the importance of normal occlusion and of maintaining the full complement of teeth in the consideration and treatment of cases of malocclusion, but I feel that I would not be true to my trust in the science I am trying to

Fig. 43.

uphold if I closed without showing you at least a glimpse of the baneful effects of mutilation by extraction.

Fig. 43 shows the effects of sacrificing all four first molars at the age of nine years for the purpose of preventing malocclusion, and you see how successful the effort was. Not only have the teeth that remained been rendered almost useless for mastication, but in recent years there has been chronic pericementitis, due to the pressure of the malocclusion on the molars in their tipped and abnormal positions. The facial lines were also greatly marred by the arrest in the development of the alveolus, for without the wedging influence of those most important teeth, the first molars, the teeth anterior could not be pushed forward by the development and eruption of the second and third molars to properly contour the face.

Fig. 44.

And this condition is not peculiar to this case, but is the result in all cases where the first molars have been sacrificed. I have carefully examined many hundreds of such cases where one or more of the first molars has been sacrificed, and I have yet to see a single instance where it was not followed by malocclusion similar to this.

Gold-capping of the leaning molars was resorted to in this case to improve the occlusion—only to aggravate the condition, for the gold crowns only made a longer leverage for the occlusion to act upon in tipping the teeth.

There seemed to me but one rational plan of treatment,—namely, to gain the space lost by the molars and then have them replaced by artificial substitutes. Fig. 44 shows all the teeth anterior to the space to have been carried forward and those posterior to have been tipped backward to approximately their correct positions, and the case ready for the bridge- or plate-maker.

A point of much interest in connection with this case is that, although the patient was thirty-eight years old, the teeth moved as rapidly and as easily as they ordinarily do in patients eighteen years of age.

Fig. 45.

I believe that if there were more skilful orthodontists to whom patients might be referred previous to having bridges inserted in their mouths, far better results would often follow the bridging process. The placing of bridges upon leaning piers seems to be as unmechanical as it is unnecessary, and the dentist who will invent a practical bridge for the restoration of lost first molars shall be called blessed, and there is a great future before him.

Fig. 45 shows the face before treatment, and you will note how greatly it is lacking in proper contour in the region of the mouth, and how greatly it has been improved in Fig. 46, which shows it at the completion of treatment.

Fig. 46.

I wish that I might have time to show you at least fifty slides bearing upon mutilation of the dental apparatus by extraction, but as it is late I will let this remarkable case, which, as I have said, is typical in its results, suffice.

In conclusion, let me say I have touched but a few of what seem to me the important places in orthodontia. Each class, division, and subdivision is ample for a full evening's discussion, but if I have awakened a higher appreciation of occlusion and convinced you that the first molar tooth is not only first in importance but first to correct if in malposition, I will have accomplished much, but no less than if you have been brought to realize that the hasty, ruthless sacrifice of teeth for the correction or prevention of malocclusion is as barbarous and unscientific as it is disastrous in its results. If I have done this I shall always feel that my mission to this society has been an enjoyable and fruitful one.

## Figures and Tables

**Fig. 1. f1:**
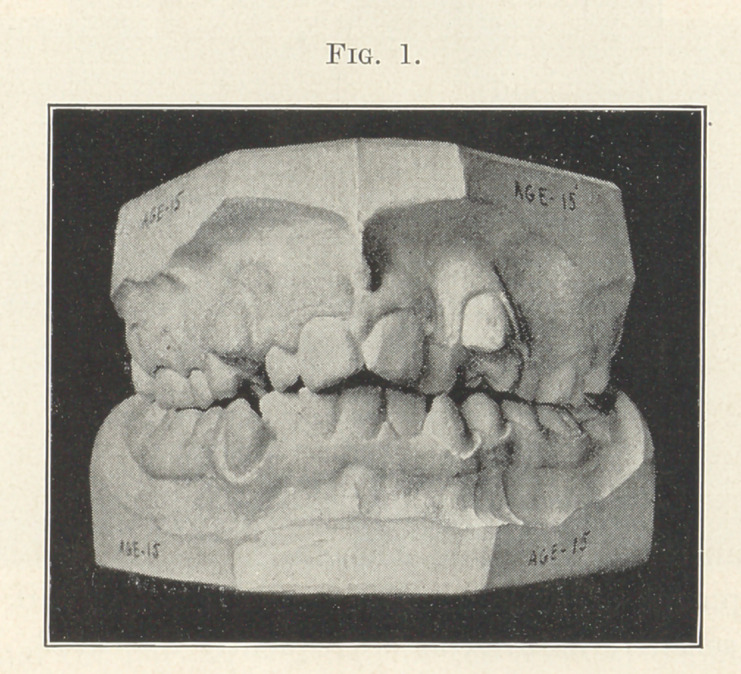


**Fig. 2. f2:**
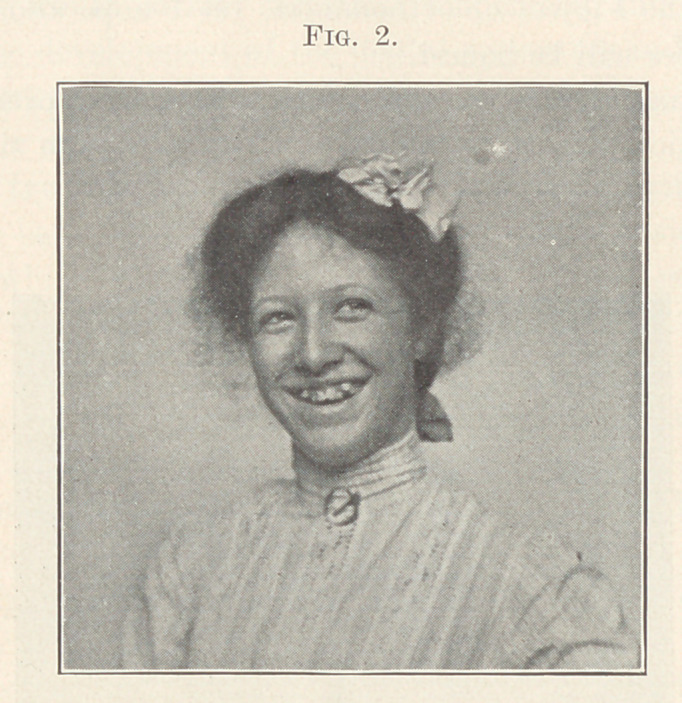


**Fig. 3. f3:**
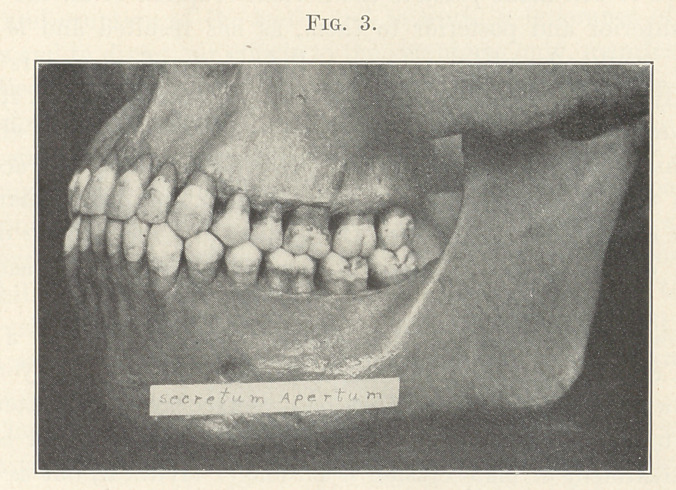


**Fig. 4. f4:**
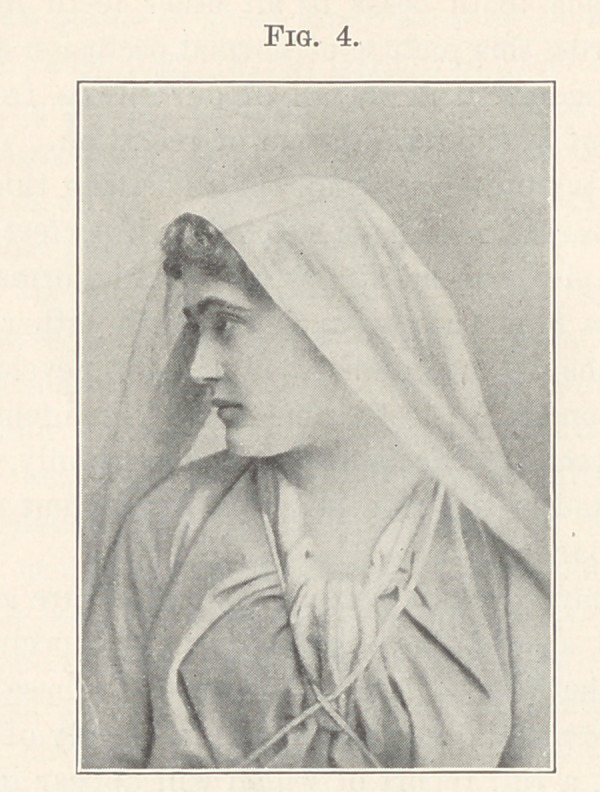


**Fig. 5. f5:**
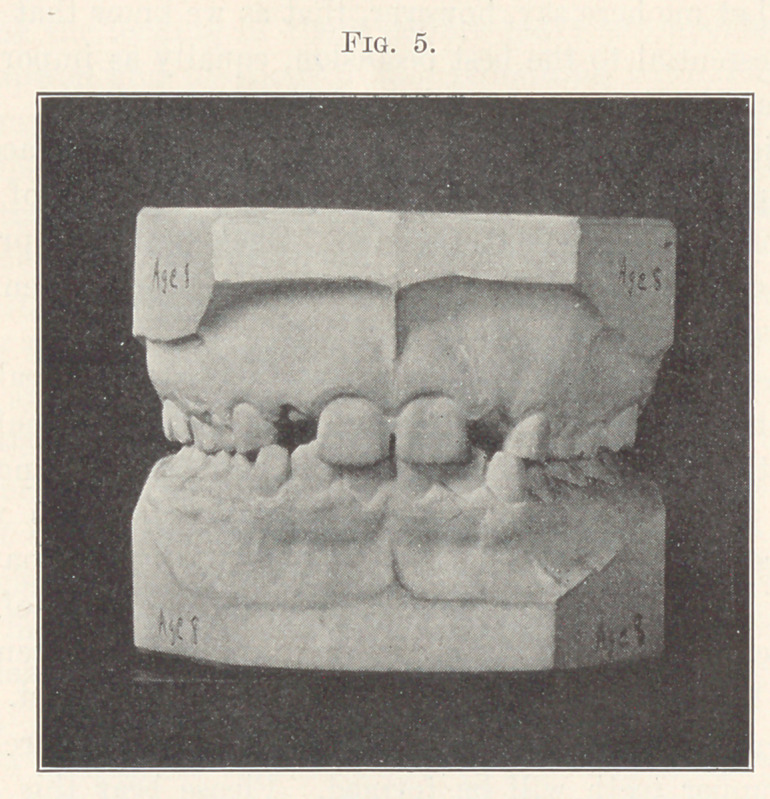


**Fig. 6. f6:**
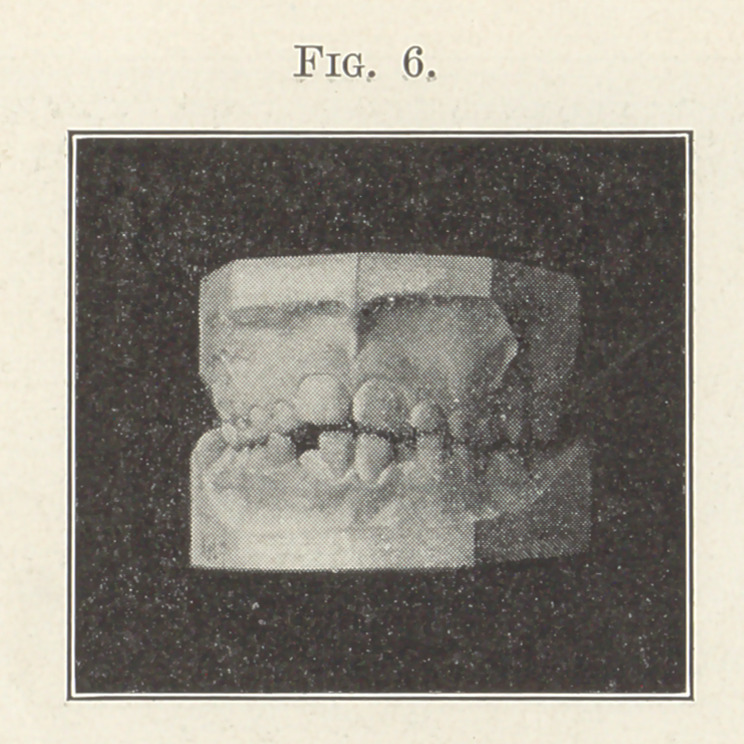


**Fig. 7. f7:**
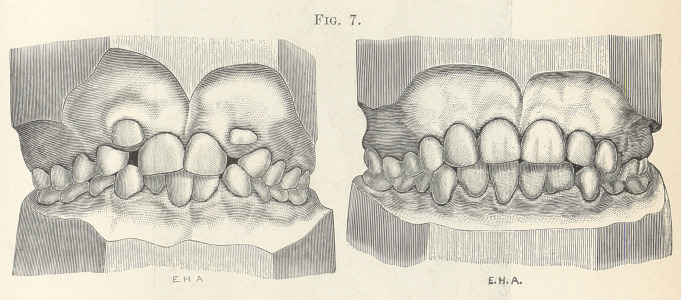


**Fig. 8. f8:**
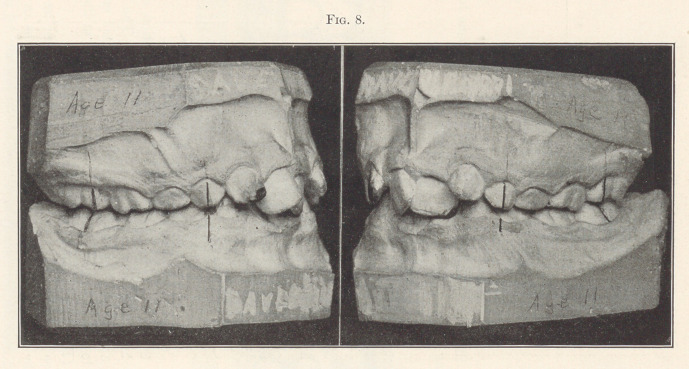


**Fig. 9. f9:**
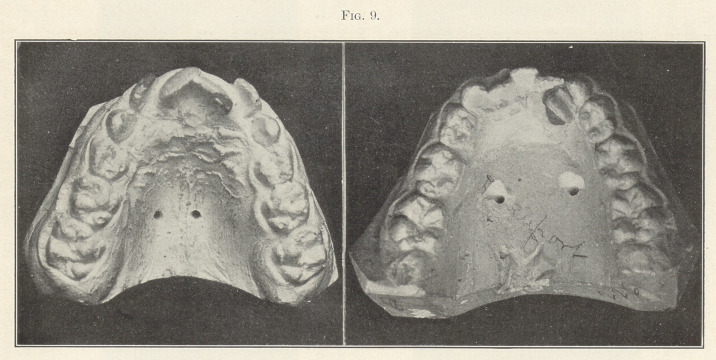


**Fig. 10. f10:**
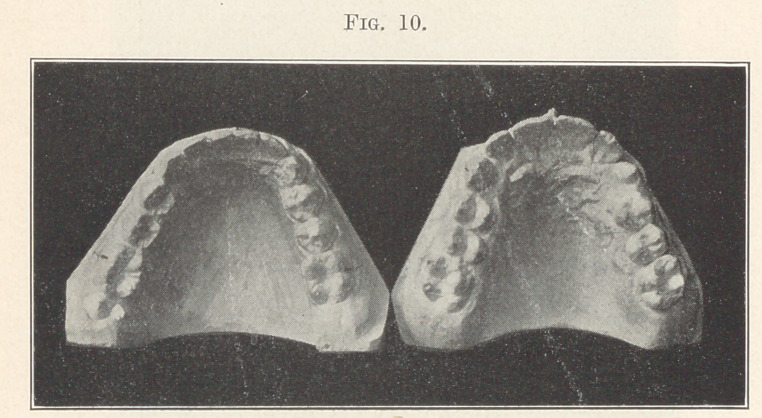


**Fig. 11. f11:**
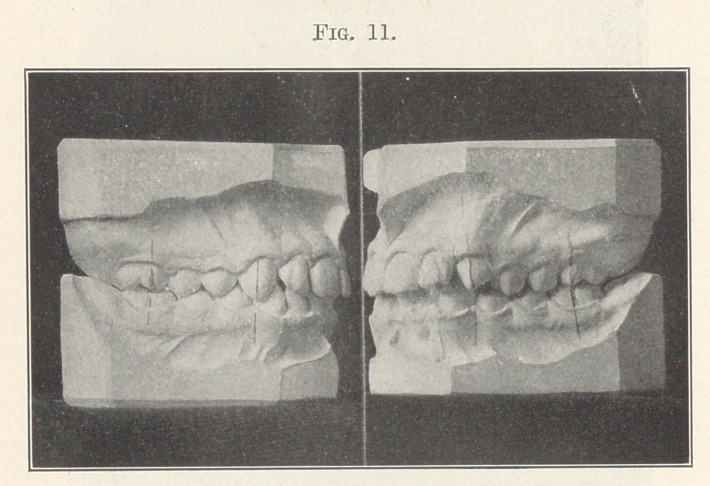


**Fig. 12. f12:**
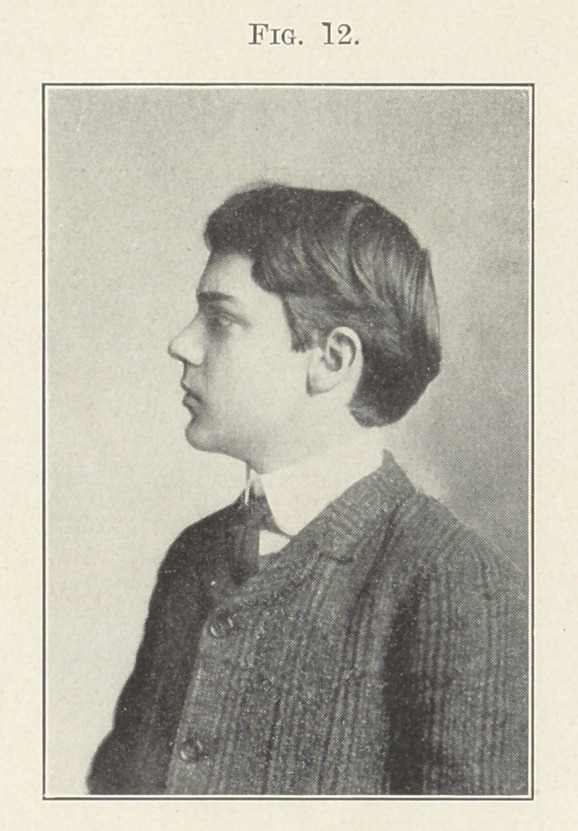


**Fig. 13. f13:**
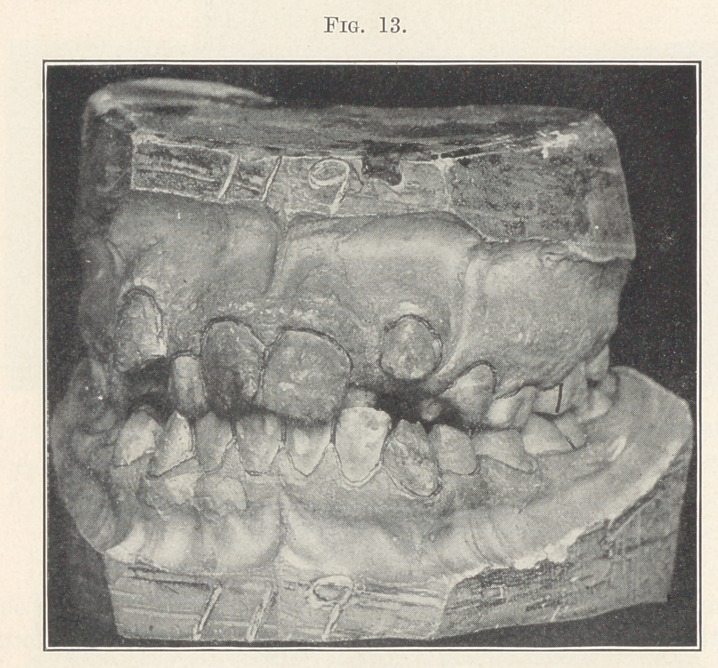


**Fig. 14. f14:**
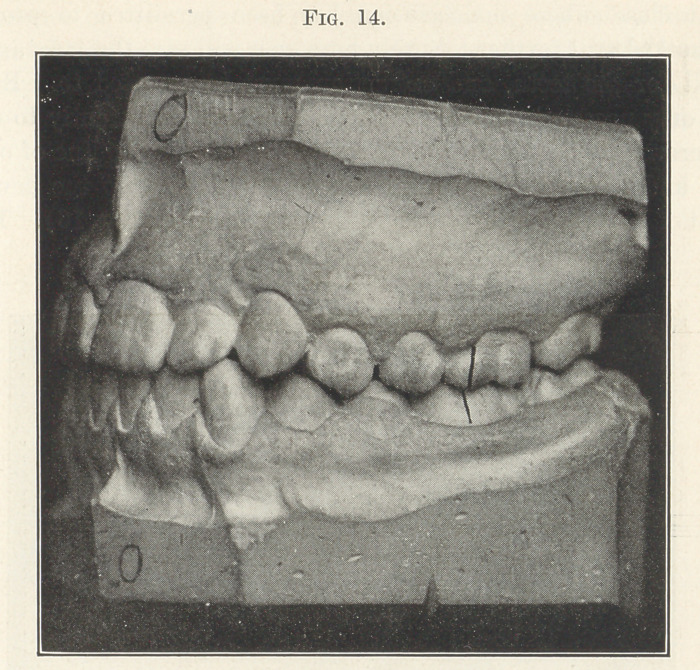


**Fig. 15. f15:**
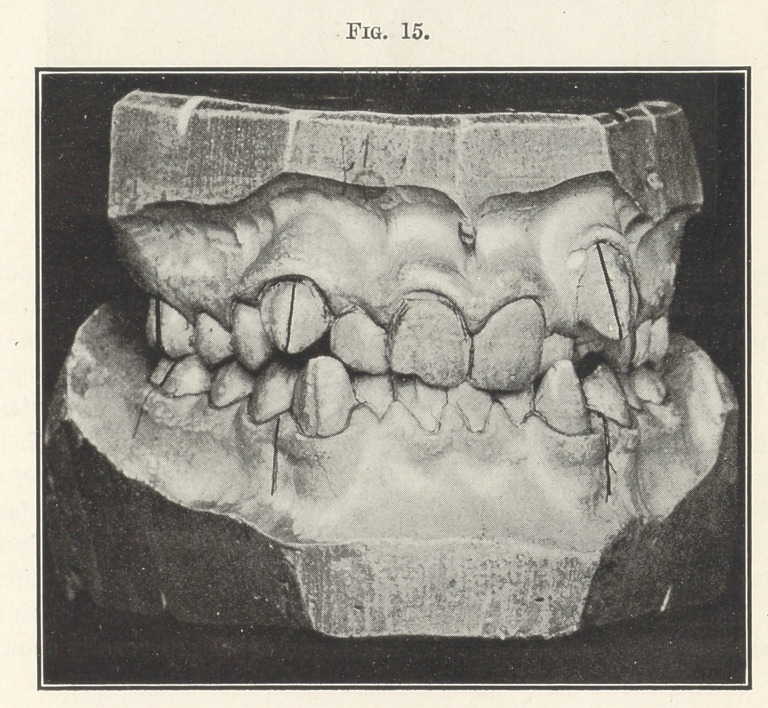


**Fig. 16. f16:**
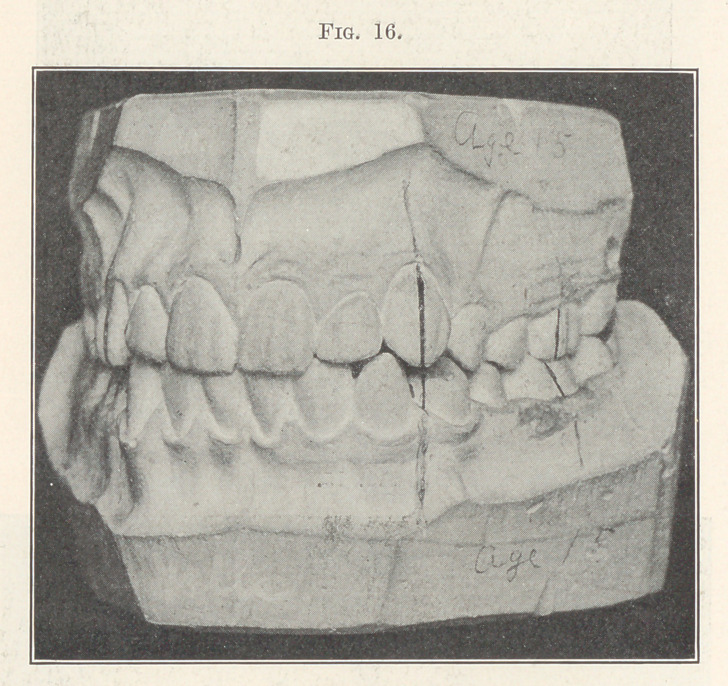


**Fig. 17. f17:**
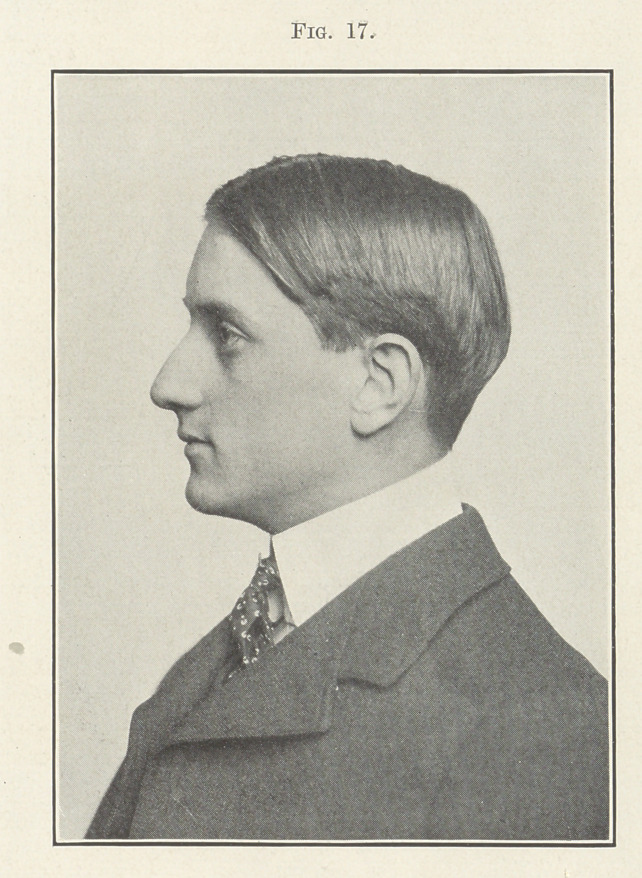


**Fig. 18. f18:**
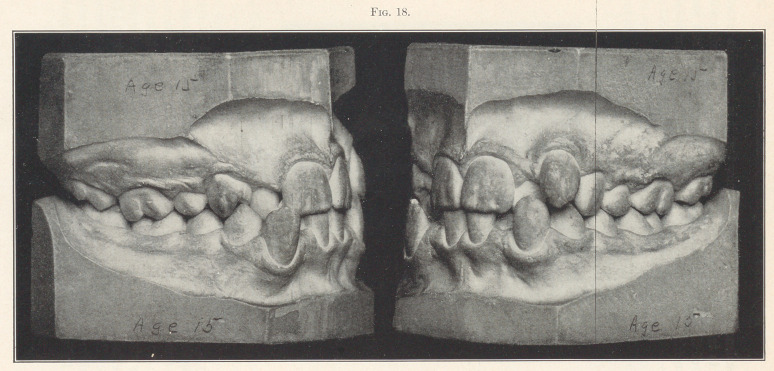


**Fig. 19. f19:**
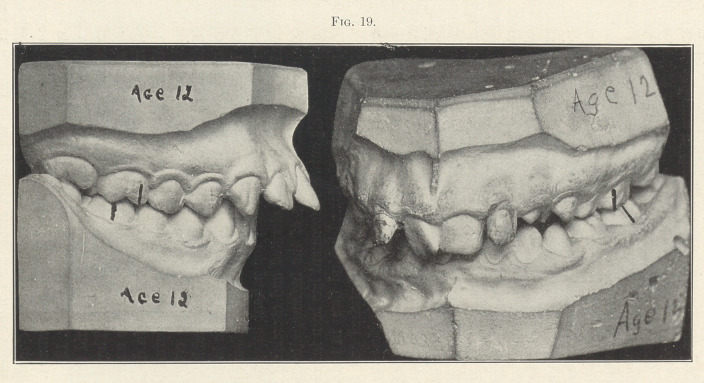


**Fig. 20. f20:**
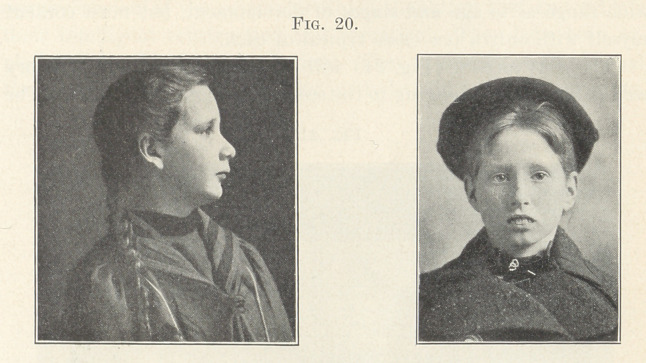


**Fig. 21. f21:**
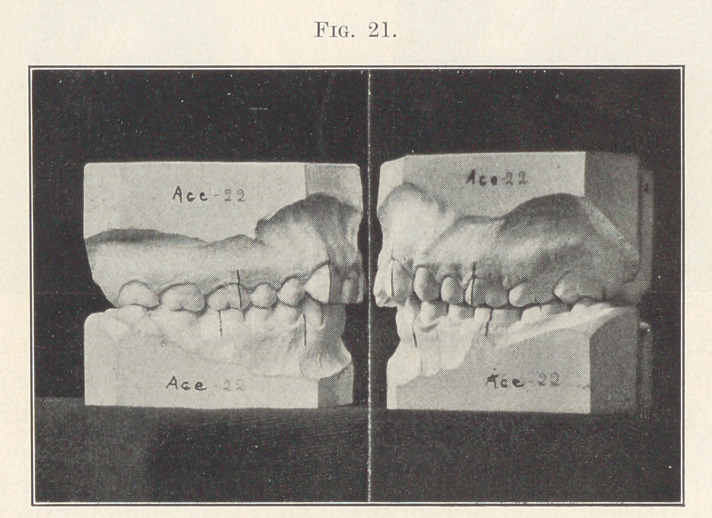


**Fig. 22. f22:**
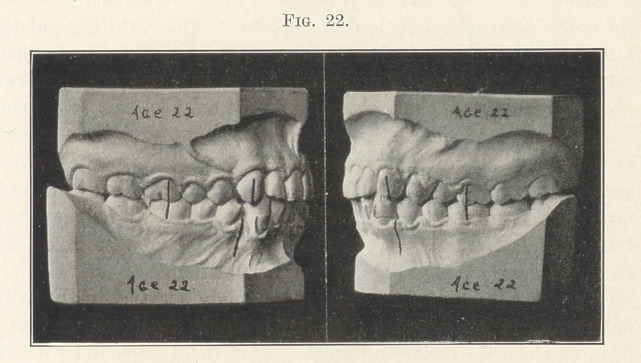


**Fig. 23. f23:**
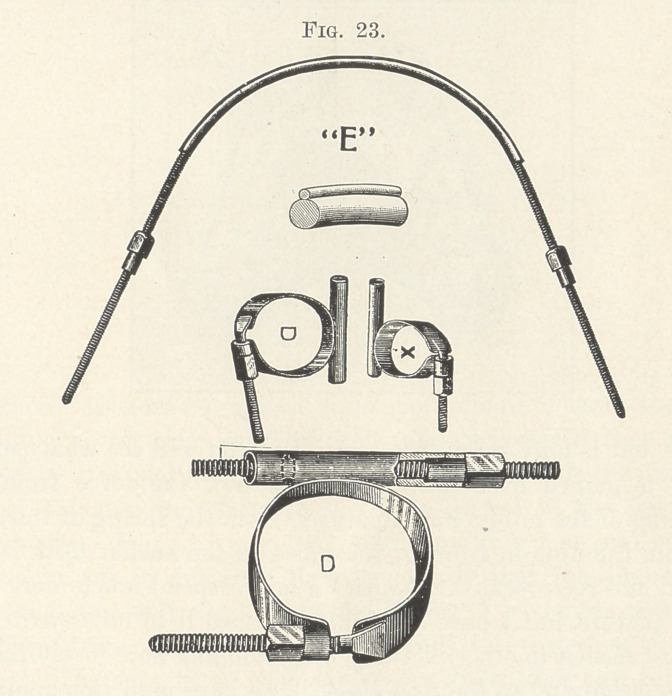


**Fig. 24. f24:**
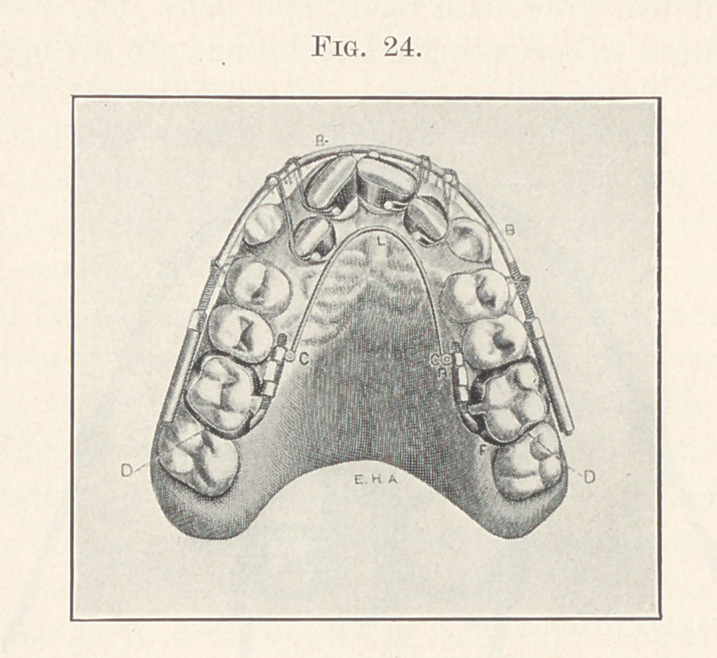


**Fig. 25. f25:**
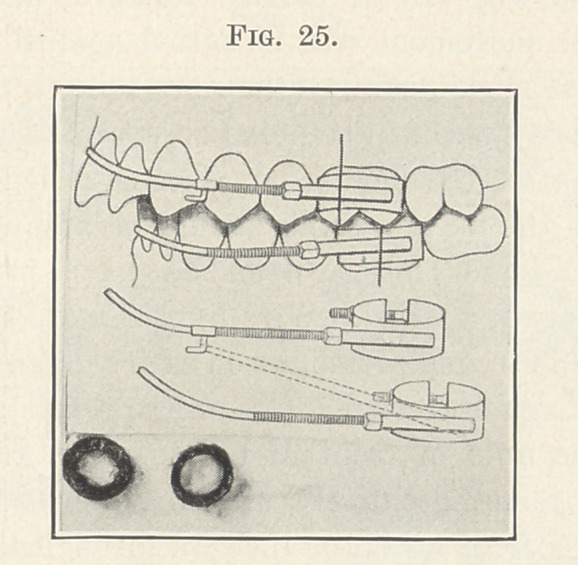


**Fig. 26. f26:**
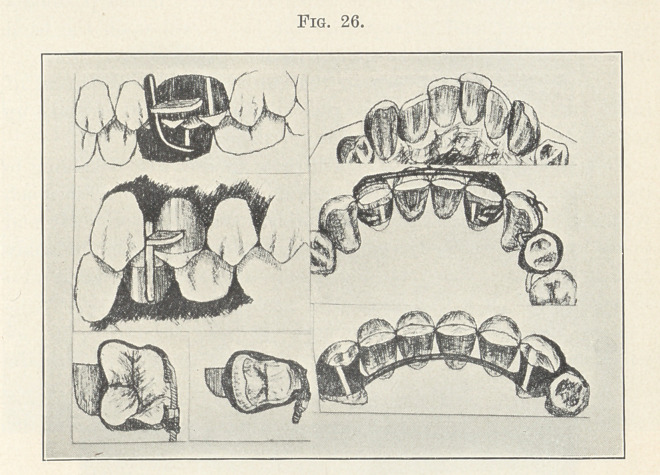


**Fig. 27. f27:**
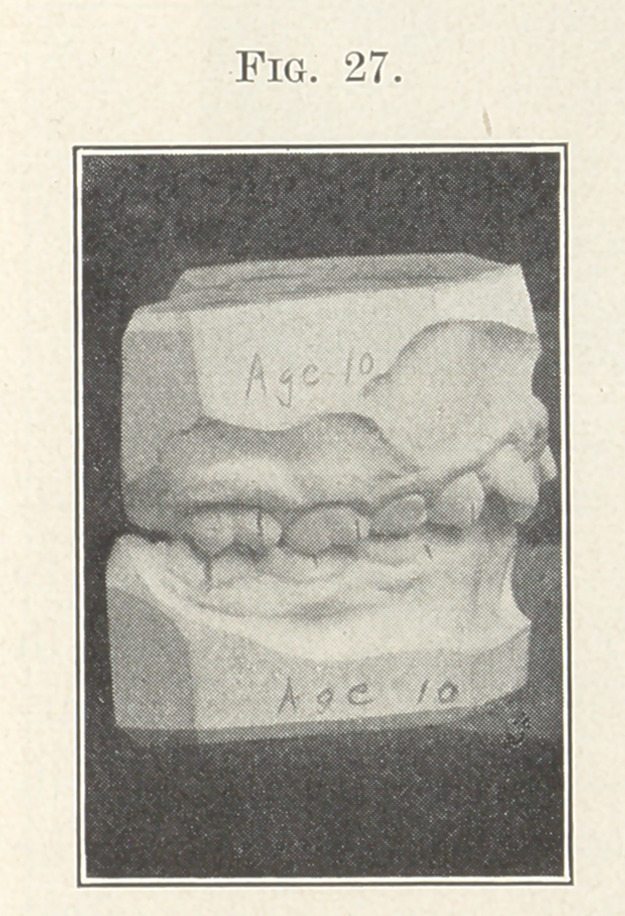


**Fig. 28. f28:**
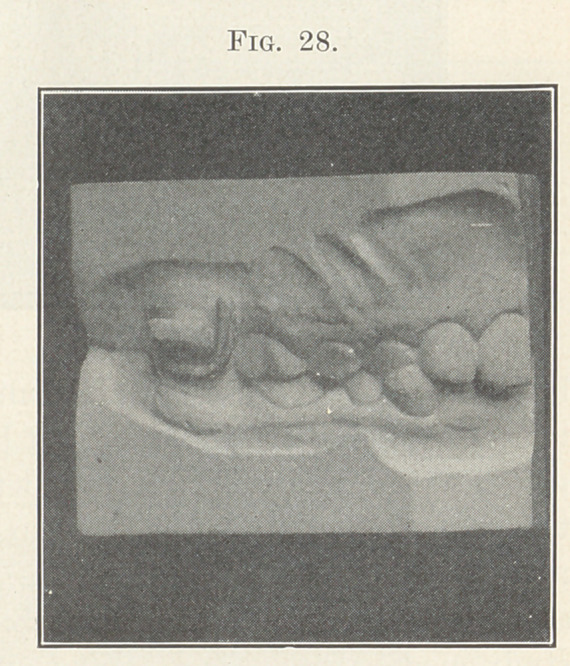


**Fig. 29. f29:**
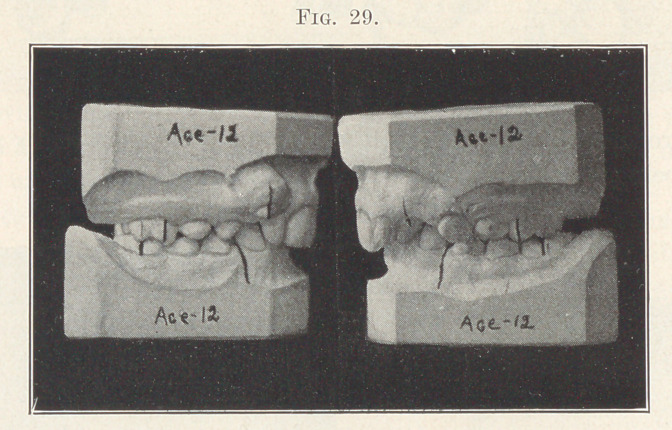


**Fig. 30. f30:**
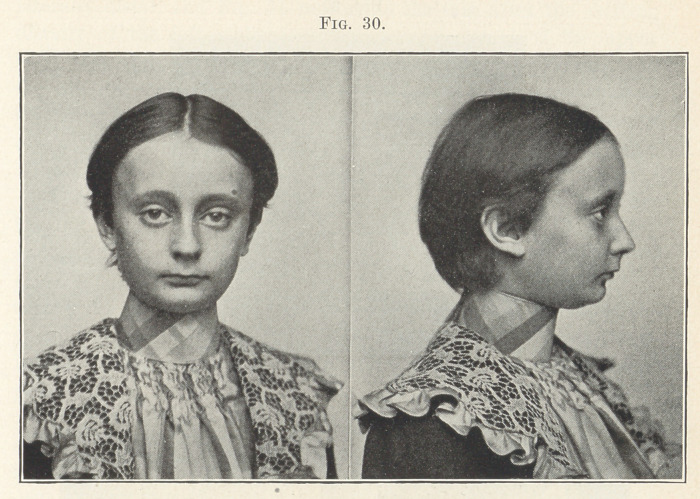


**Fig. 31. f31:**
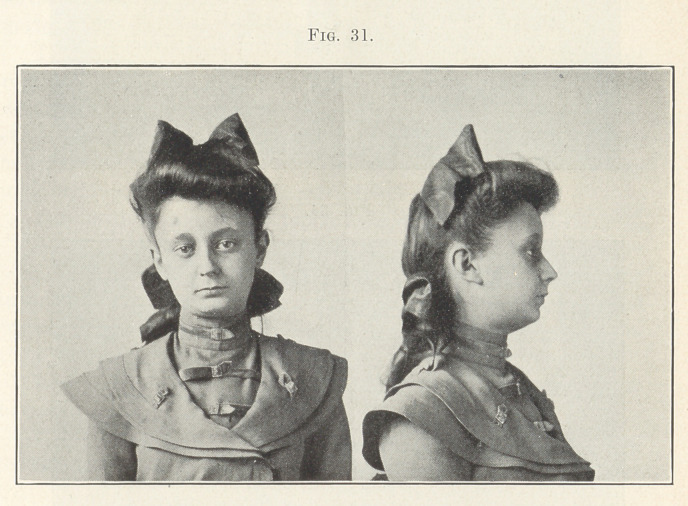


**Fig. 32. f32:**
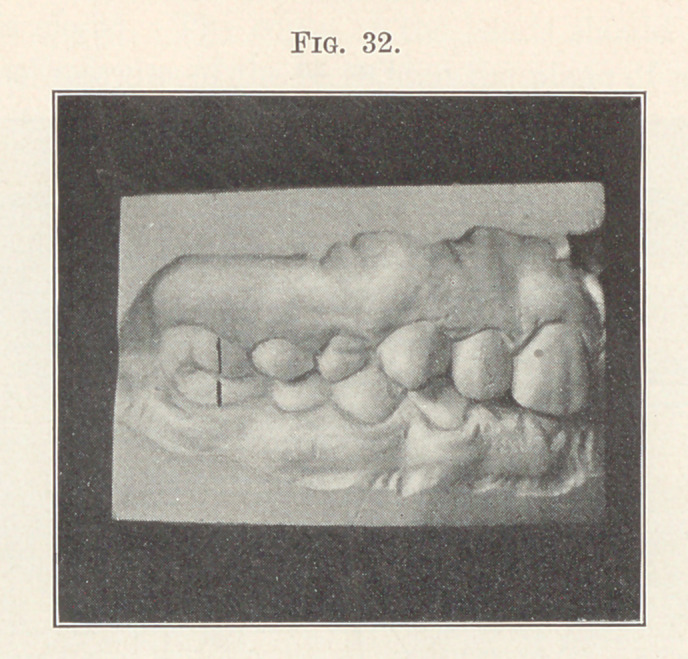


**Fig. 33. f33:**
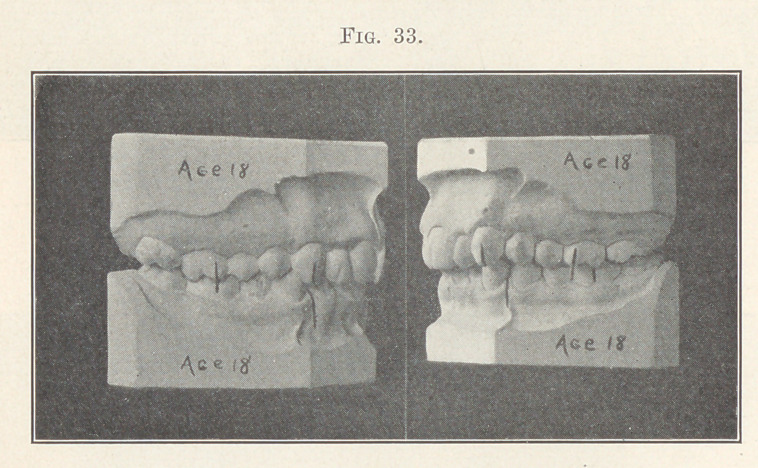


**Fig. 34. f34:**
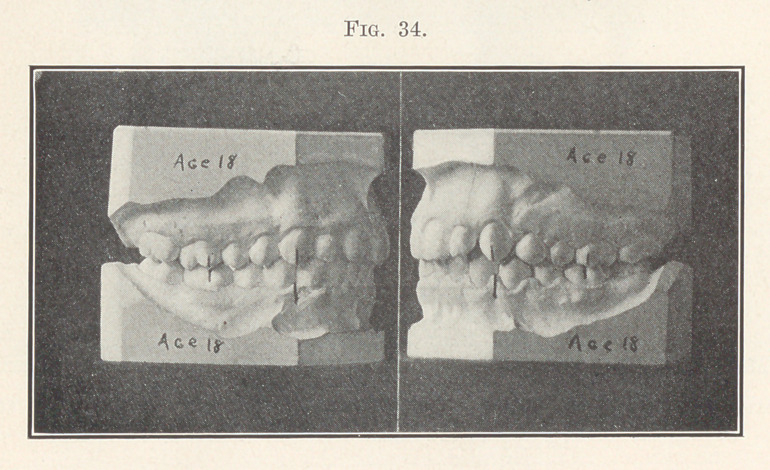


**Fig. 35. f35:**
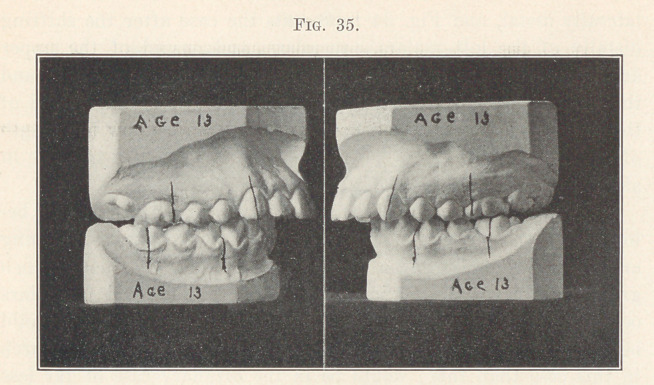


**Fig. 36. f36:**
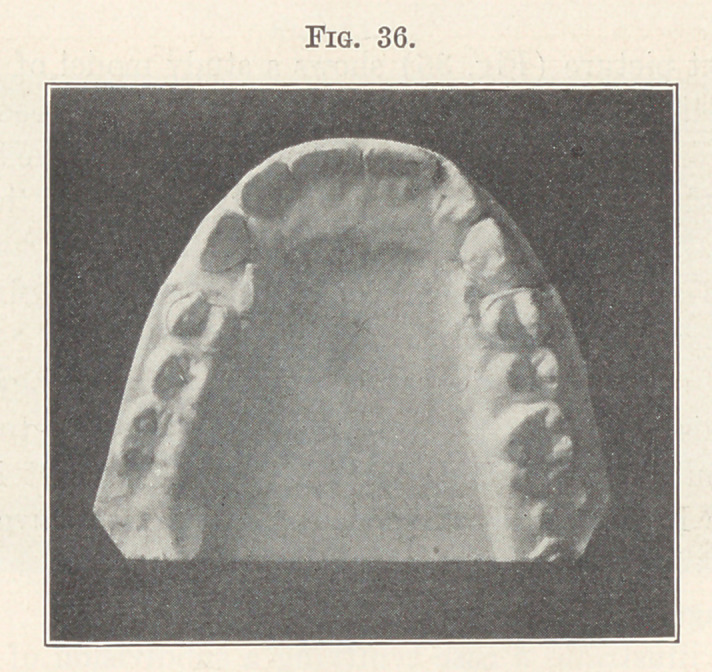


**Fig. 37. f37:**
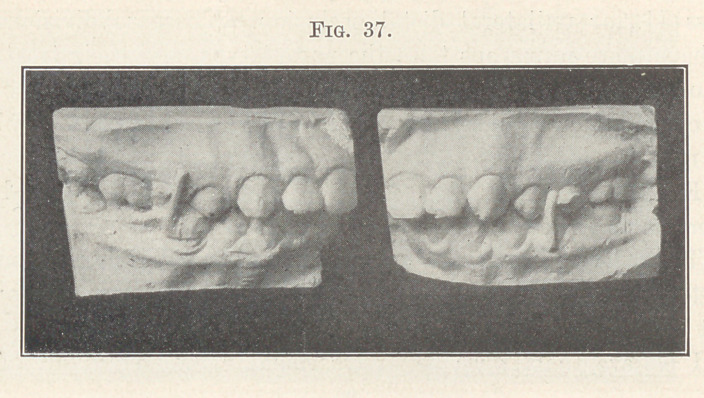


**Fig. 38. f38:**
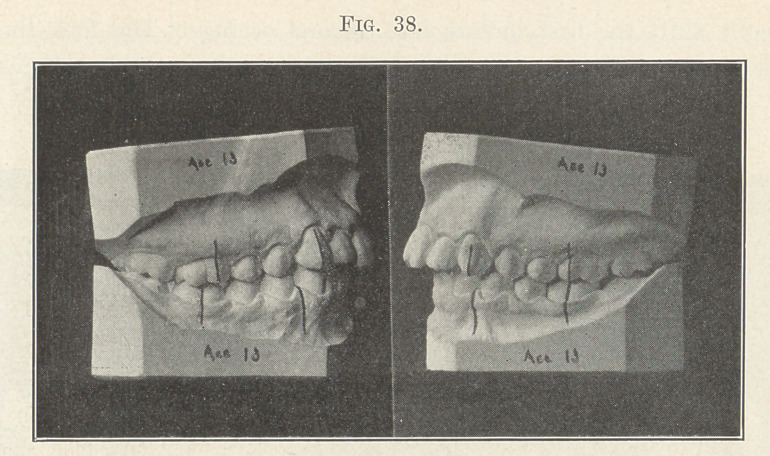


**Fig. 39. f39:**
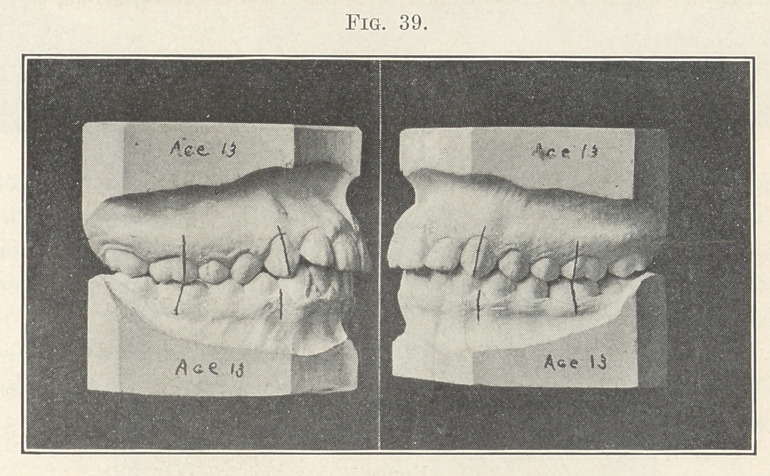


**Fig. 40. f40:**
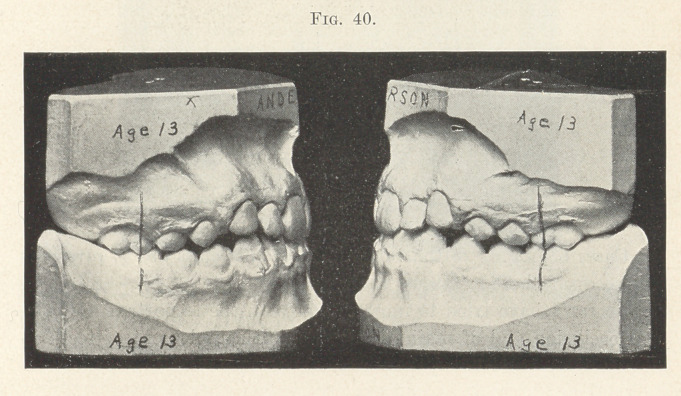


**Fig. 41. f41:**
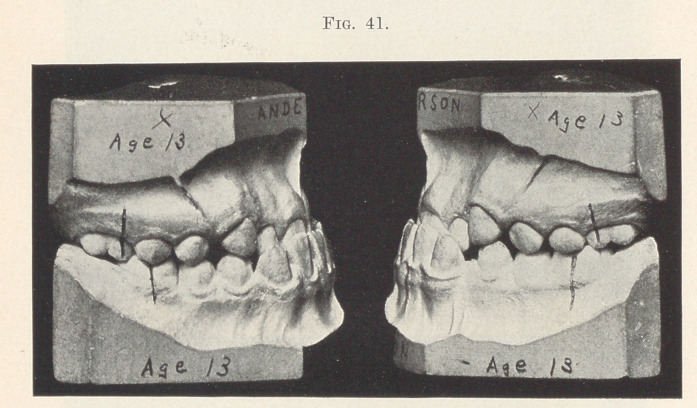


**Fig. 42. f42:**
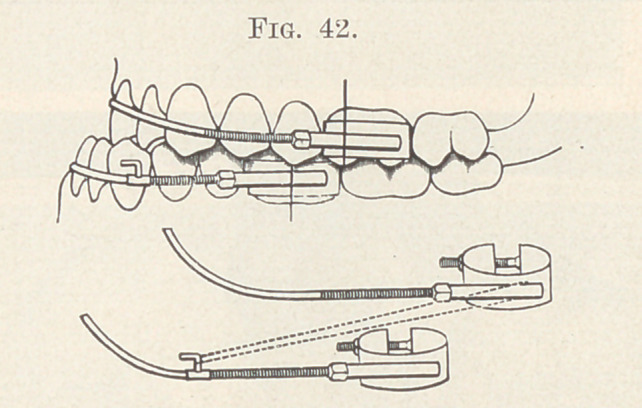


**Fig. 43. f43:**
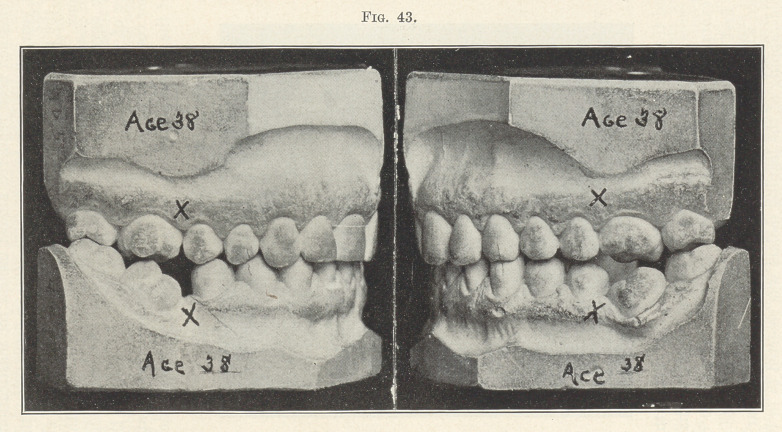


**Fig. 44. f44:**
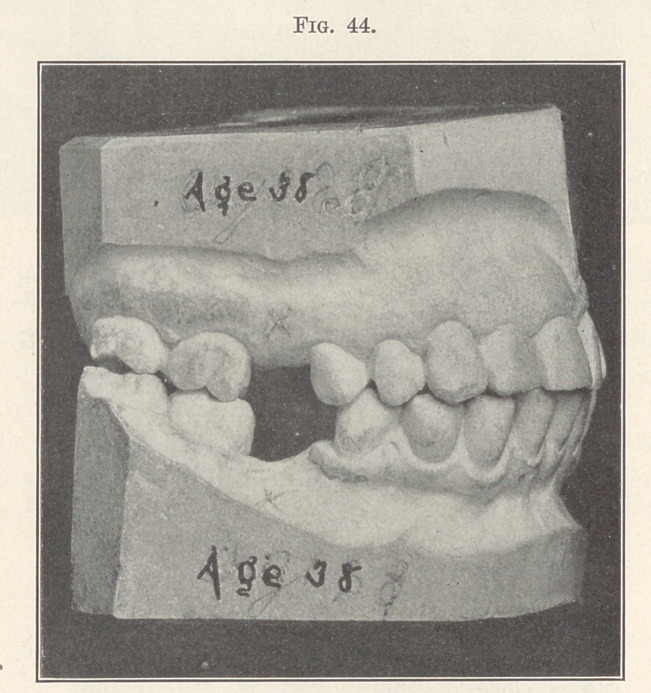


**Fig. 45. f45:**
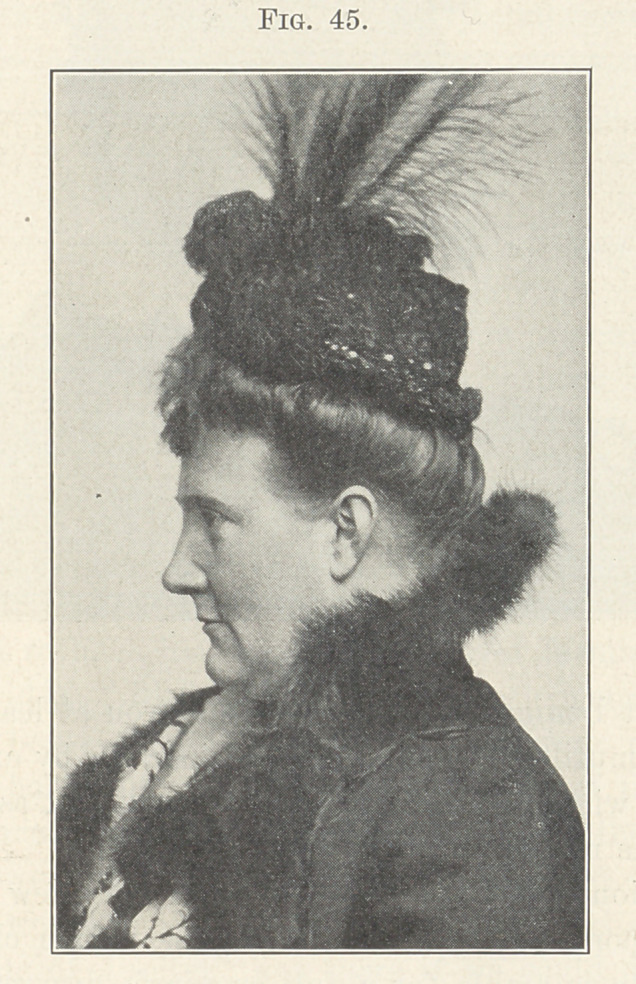


**Fig. 46. f46:**